# Cannabidiol is an effective helper compound in combination with bacitracin to kill Gram-positive bacteria

**DOI:** 10.1038/s41598-020-60952-0

**Published:** 2020-03-05

**Authors:** Claes Søndergaard Wassmann, Peter Højrup, Janne Kudsk Klitgaard

**Affiliations:** 1 Department of Biochemistry and Molecular Biology, Research Unit of Molecular Microbiology, University of Southern Denmark, Odense, Denmark; 2 Department of Biochemistry and Molecular Biology, Protein Research Group, University of Southern Denmark, Odense, Denmark; 3 Institute of Clinical Research, Research Unit of Clinical Microbiology, University of Southern Denmark, Odense, Denmark

**Keywords:** Drug discovery, Microbiology, Antimicrobials

## Abstract

The cannabinoid cannabidiol (CBD) is characterised in this study as a helper compound against resistant bacteria. CBD potentiates the effect of bacitracin (BAC) against Gram-positive bacteria (*Staphylococcus* species, *Listeria monocytogenes*, and *Enterococcus faecalis*) but appears ineffective against Gram-negative bacteria. CBD reduced the MIC value of BAC by at least 64-fold and the combination yielded an FIC index of 0.5 or below in most Gram-positive bacteria tested. Morphological changes in *S. aureus* as a result of the combination of CBD and BAC included several septa formations during cell division along with membrane irregularities. Analysis of the muropeptide composition of treated *S. aureus* indicated no changes in the cell wall composition. However, CBD and BAC treated bacteria did show a decreased rate of autolysis. The bacteria further showed a decreased membrane potential upon treatment with CBD; yet, they did not show any further decrease upon combination treatment. Noticeably, expression of a major cell division regulator gene, *ezrA*, was reduced two-fold upon combination treatment emphasising the impact of the combination on cell division. Based on these observations, the combination of CBD and BAC is suggested to be a putative novel treatment in clinical settings for treatment of infections with antibiotic resistant Gram-positive bacteria.

## Introduction

Since the discovery of penicillin in 1928 by Sir Alexander Fleming, antibiotics have saved millions of lives from fatal infections world-wide. However, with time bacteria have developed mechanisms to escape the effects of antibiotics. The amount of antibiotics used seems to be directly related to development of antibiotic resistance. Similarly, a growing number of multi drug resistant (MDR) bacteria is a result of inadequate intentions to solve the resistance problem and increasing unmet demands for new antibacterial drugs^1^.

With fewer antibiotics available to treat MDR bacterial infections, the possibility of entering a pre-antibiotic era is looming ahead. Alternative strategies are being explored and helper compounds, also known as antibiotic potentiators or resistant breakers, are attracting attention^2^. Helper compounds are non-antibiotic compounds functioning as adjuvants for antibiotics to operate in synergy through various mechanisms including efflux pump inhibition, inhibition of enzymes, and changes in membrane permeability, all of which may contribute to increasing the efficacy of a specific antibiotic ^3,4^. Drugs found to contain helper compound properties are normally used for treatment of non-infectious diseases but may contain some degree of antibacterial activity itself^5^. Helper compounds are usually associated with side-of-action in the central and peripheral nervous system as local anaesthetics and in psychopharmaceutic practice, where they usually block membrane associated transporter activity^5^.

Overuse of antibiotics is the main cause of antibiotic resistance. Therefore, by combining an antibiotic with a helper compound less antibiotic is needed in order to achieve bacterial growth inhibition or killing compared to using the antibiotic alone. This strategy may therefore decrease the likelihood of resistance development, and investigations to identify efficient helper compounds are thus important.

Cannabinoids are categorised as either endogenous cannabinoids, which are cannabinoids produced by the human body, or exogenous cannabinoids, which are produced either by plants such as *Cannabis sativa* or synthetically. Cannabinoids act on the endocannabinoid system of the human body^6^ consisting of two G-protein coupled receptors (GPCR). These are named cannabinoid type 1 and 2 (CB1 and CB2) receptors, and, depending on the specific cannabinoid, the binding results in either an agonistic or antagonistic downstream effect^7^. Besides endocannabinoids being ligands for the endocannabinoid receptors, exogenous cannabinoids are also ligands for the receptors. One of the best characterised exogenous ligands is tetrahydrocannabinol (THC). It is a partial agonist for both CB1 and CB2 receptor mediating effects such as analgesia, muscle relaxation, and antiemetic effects, but also results in negative effects such as anxiety, psychosis, and sedation. Another exogenous cannabinoid is cannabidiol (CBD), which has been observed to decrease the adverse negative effects of THC. CBD is an antagonist of both CB1 and CB2 receptor leading to anti-sedative, anti-psychotic, and anxiolytic effects^7^. However, these are not the only known effects of CBD, as it is able to cause a variety of different effects such as inhibition of cancer cell growth^8^, neuroprotection in both neuro-degenerative diseases such as Parkinson’s Disease^9^ and post-ischemia^10^, and anti-inflammatory effects as in type-1 diabetes^11^. Not much is known regarding antimicrobial effects of cannabinoids and even less on the mechanism of action. Endocannabinoids and exogenous cannabinoids such as CBD have been observed to inhibit growth of bacteria^12–14^, yet the use of cannabidiol as an antibiotic adjuvant has not been studied so far.

In the present study, we aim to characterise cannabidiol as a potential helper compound against resistant bacteria in combination with the cyclic peptide antibiotic bacitracin (BAC). BAC is a mixture of related cyclic peptides operating as a bactericidal antibiotic by interfering with the cell wall and interrupting the biosynthesis of the peptidoglycan leading to cell lysis^15^.

## Results

### The combination of CBD and BAC is effective against Gram-positive bacteria

Initially, we validated the antimicrobial effect of cannabidiol (CBD) against the Gram-positive bacterium Methicillin-Resistant *Staphylococcus aureus* (MRSA) as previously published by Appendino and colleagues^14^ but also for *Enterococcus faecalis* (*E. faecalis*)*, Listeria monocytogenes* (*L. monocytogenes*), and Methicillin-Resistant *Staphylococcus epidermidis* (MRSE). We found the Minimum Inhibitory Concentration (MIC) to be 4 µg/mL for *S. aureus*, *L. monocytogenes*, and the MRSE strain and 8 µg/mL for *E. faecalis*, indicating that Gram-positive bacteria are sensitive towards CBD (Table 1).

**Table 1:** *FIC index indicates synergy when FIC index ≤ 0.5, indifference when 0.5 < FIC index < 4, and antagonism when FIC index > 4^37^.

Strain	MIC CBD (µg/mL)	MIC BAC (µg/mL)	Fold reduction in MIC of BAC when combined with ½xMIC CBD	FIC Index*
MRSA USA300 FPR3757	4	64	32–64	0.5
*E. faecalis* (13–327129)	8	64	≥64	0.375
*L. monocytogenes* (EGD)	4	512	8	0.625
MRSE (933010 3F-16 b4)	4	32	64	0.5

To determine whether CBD would induce a higher susceptibility of BAC in Gram-positive bacteria, MICs of BAC were determined for the four Gram-positive bacteria in the presence of CBD. Remarkably, the MIC of BAC was decreased by 8 to at least 64-fold when combined with 1/2 x MIC of CBD compared to MIC of BAC alone in the different Gram-positive strains (Table 1). Furthermore, the Fractional Inhibitory Concentration (FIC) index was determined for each Gram-positive bacteria. The results showed a FIC index at 0.5 for both MRSA USA300 and MRSE and 0.375 for *E. faecalis* indicating weak synergistic effect between the compounds CBD and BAC (Table 1). After combining CBD with other antibiotics, both similar and different types, we concluded that CBD had the best effect together with BAC (see Supplementary Figure S1).

**Figure 1:**
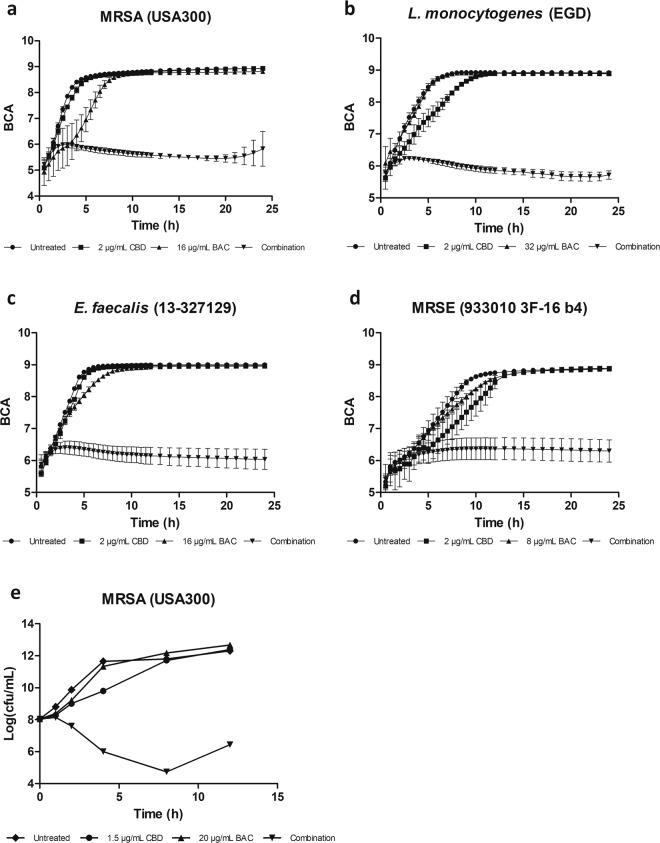
Growth curves of cannabidiol (CBD) in combination with bacitracin (BAC). Bacterial density (BCA: Background corrected absorption) was measured using an oCelloScope for 24 hours; (**a**) Methicillin-resistant *Staphylococcus aureus* USA300 FPR 3757, (**b**) *Enterococcus faecalis* (13-327129), (**c**) *Listeria monocytogenes* EGD, (**d**) Methicillin-resistant *Staphylococcus epidermidis*. (**e**) Time-kill assay showing the effect of CBD and BAC in monotherapy and combination in MRSA USA300 FPR3757. Viability was monitored by OD_600_ readings and CFU/mL determinations. Growth experiments were repeated at least three times and the time-kill assay was repeated twice with similar results.

To assess the potentiating effect of CBD on BAC over time, measurements of bacterial growth over 24 hours in the presence of either CBD alone or in combination with BAC were performed. The assessment concentrations of CBD were at 2 µg/mL and 8, 16, and 32 µg/mL for BAC. As seen in Fig. 1a, growth of *S. aureus* is inhibited by 2 µg/mL CBD and 16 µg/mL BAC combined compared to monotherapies of the individual compounds. The results suggest that CBD can potentiate the antimicrobial effects of BAC. Similarly, growth measurements of *E. faecalis*, MRSE, and *L. monocytogenes* on monotherapies and combination (Fig. 1b–d), suggests that the combination of CBD and BAC is useful against other Gram-positive bacteria.

To clarify whether CBD and BAC act in synergy, time-kill assays were performed (Fig. 1e). CBD and BAC reduced the viability by 6 log_10_ CFU/mL compared to CBD alone. The result shows that a clear synergistic effect in fact exists between CBD and BAC, and that the effect is bactericidal. The slight re-initiation of growth after 8 hours is almost certainly caused by degradation or oxidation of the cannabinoid^16^. To verify that the decreased CFU upon combination treatment is caused by killing of the bacteria and not due to clustering of the cells, microscopy was performed at the time 1, 2, 4, and 8 hours post treatment (Supplementary Figure S2). Images show no additional clustering of the cells treated with the combination compared to the other treatments.

To further assess the spectrum of use for the combination of CBD and BAC, growth of Gram-negative bacteria upon treatment was measured as well. The Gram-negative bacteria tested were strains of *Pseudomonas aeruginosa*, *Salmonella typhimurium*, *Klebsiella pneumoniae*, and *Escherichia coli* (Supplementary Figure S3). Experiments for CBD and BAC against the Gram-negative bacteria revealed MIC values above 128 µg/mL for all tested bacteria, presumably due to the outer membrane. In addition, the experiments did not reveal any synergy between CBD and BAC in the concentrations tested, limiting the use of the combination to Gram-positive bacteria.

### CBD and BAC causes morphological changes

**Figure 2:**
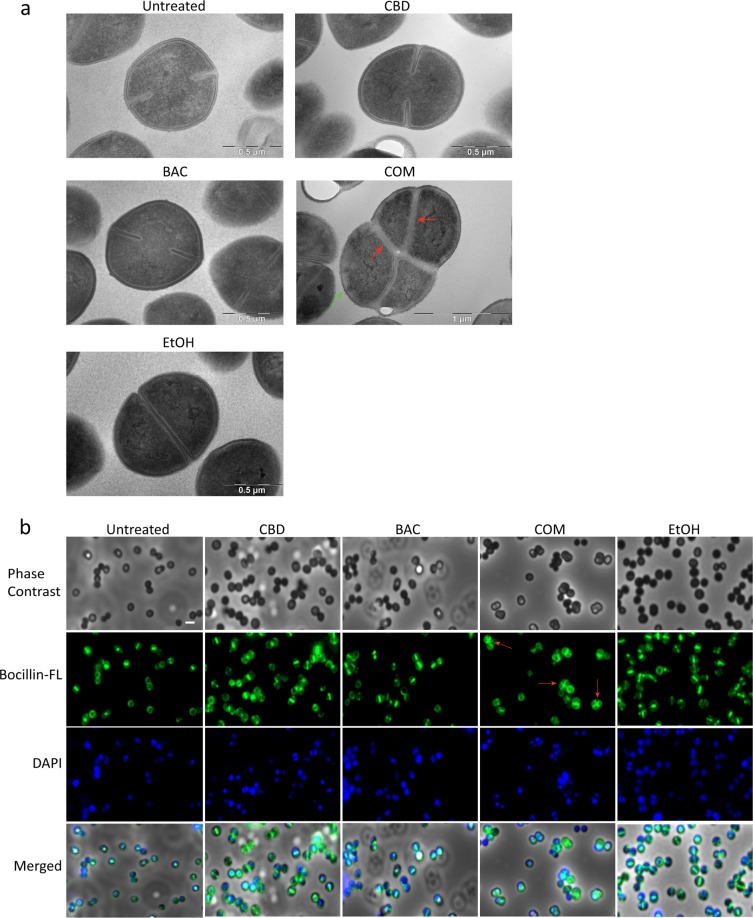
Morphology of USA300 FPR3757 following treatment with CBD and/or BAC. Cultures were subjected to the drugs for 2.5 hours as described in Methods. (**a**) Morphology imaged by transmission electron microscopy. TEM overview images are shown in Supplementary Figure S4. (**b**) Bocillin-FL labelled PBPs visualised using Olympus IX83 fluorescence microscope. Images merged with DAPI stain solution to localise PBPs in the bacteria. Defects in cell division are marked by red arrows and irregularities around cell envelope are marked by a green arrow.

We have established that CBD can potentiate the effect of BAC in Gram-positive bacteria. The next step is to study the mechanism underlying this synergy. First, we looked at the morphological changes of *S. aureus* USA300 upon exposure to CBD and/or BAC by treating a culture at start exponential phase for 2.5 hours and then performing transmission electron microscopy (TEM) of the cells. Results showed that CBD and BAC alone caused no morphological changes, as they resembled untreated control and EtOH control. However, as seen in Fig. 2a, treatment with the combination of CBD and BAC resulted in large undivided cells with several septa formations or several initiations of septum formation indicating severe defects in cell division (red arrows) and irregularities around cell envelope (green arrow) (Supplementary Figure S4). The result was confirmed by staining the Penicillin Binding Proteins (PBPs) in the membrane using Bocillin-FL, a fluorescence-conjugated penicillin V derivative (Fig. 2b) which showed similar morphology (red arrows). As peptidoglycan synthesis occurs both at the septal and peripheral cell wall, we can observe irregularities concerning the peptidoglycan all over the cell surface^17^. Upon exposure to either CBD or BAC alone regular septum formations were visualised, however, when treated with the combination, several septa formations appeared for some of the cells as visualised by Bocillin-FL as in the TEM images. This suggests that the combination of CBD and BAC affects the cell envelope causing irregular cell division visualised by multiple septa formations and irregular cell membrane. To study whether the cell division defect is specific for the combination of CBD and BAC, microscopy analysed using higher concentrations of CBD and BAC at 4 and 64 µg/mL, respectively, was performed (Supplementary Information Figure S5). Images show cells with multiple septa upon treatment with 64 µg/mL BAC, indicating that the effect visualised is not specific to the combination of CBD and BAC. However, it further emphasises the CBD mediated potentiation of BAC, since this phenotype did not appear at lower BAC concentration. Adding a higher concentration of CBD did not seem to cause any division defects.

### The combination of CBD and BAC decreases autolysis in *S. aureus*

**Figure 3:**
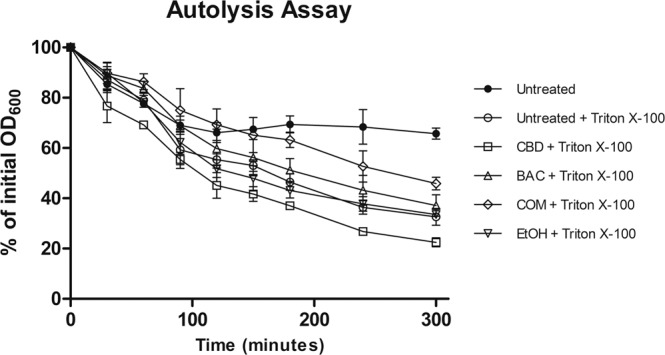
Effects of CBD and BAC on autolysis. Unstimulated and Triton X-100 stimulated autolysis of USA300 grown in BHI to early exponential phase. Statistical analysis by 2-way ANOVA with Bonferroni’s Multiple Comparison Test shows P < 0.05 when comparing Triton X-100 stimulated untreated samples with combination of CBD and BAC samples after 90 minutes, except at the 150 minutes timepoint. Detailed statistical analysis and figure including all controls are shown in Supplementary Figure S6.

Since treatment with the combination of CBD and BAC shows impaired cell division, probably causing an arrest in cell division and potentially decreased cell wall turnover, one could speculate if this would result in decreased autolysis as well. Therefore, a Triton X-100 induced autolysis assay was performed. *S. aureus* USA300 were grown until start exponential phase and stressed for one hour with CBD, BAC, CBD+BAC, EtOH or left untreated. Cells were then washed and incubated with or without triton X-100. As suspected, upon treatment with the combination of 1 µg/mL CBD and 16 µg/mL BAC a significant decreased autolysis was observed (Fig. 3) compared to the untreated control from 90 to 300 minutes except at the 150 minute timepoint, indicating cell division arrest.

### The combination of CBD and BAC does not change the cell wall composition or the degree of cross-linking

**Figure 4:**
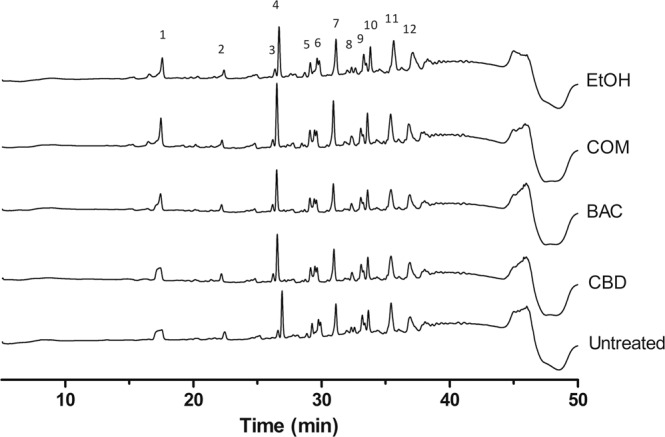
Effect of CBD and BAC on the muropeptide composition of USA300 peptidoglycan. Peptidoglycan was isolated from cultures grown to exponential phase in the absence or presence of CBD or BAC and muropeptide compositions were analysed by HPLC as described in Methods. Muropeptide analysis was performed twice with similar profiles. X-axis show retention time in minutes, Y-axis show milli absorbance units (mAU). One biological replicate is depicted in the figure. Two other replicates are shown in Supplementary Figure S7.

To further asses the irregularities around the cell envelope and the possible effect on the cell wall biosynthesis, the muropeptide composition of the peptidoglycan was analysed. Peptidoglycan was purified from *S. aureus* USA300 grown in either CBD, BAC, the combination of CBD and BAC, EtOH or left untreated and further digested using mutanolysin and analysed using HPLC. The chromatogram of purified digested muropeptides revealed the typical pattern of *S. aureus*^18^ with the highest peak found in the dimeric fraction (peak 4). Treating the bacteria with both CBD and BAC alone or in combination did not change the pattern of the HPLC chromatogram of the muropeptides (Fig. 4) indicating no change in the muropeptide composition. Even though the relative amount of some of the muropeptide fractions were significantly different, the degree of cross-linking was unaltered when compared to the untreated control (Supplementary Tables S3, S4 and S5). Based on these observations, CBD or the combination of CBD and BAC does not seem to cause changes in the cell wall composition.

### CBD causes depolarisation of the cytoplasmatic membrane

**Figure 5:**
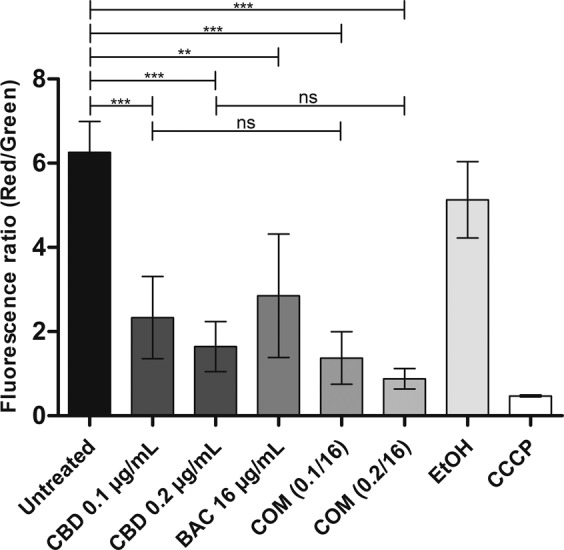
Measurements of membrane potential in USA300 treated with CBD, BAC and the combination using BacLight Bacterial Membrane Potential Kit as described in Methods. The ratio between the mean red fluorescence and mean green fluorescence was calculated as a measure of membrane potential for each sample since the dye will accumulate in unaffected cells thus emitting a red fluorescence, whereas in cells with affected membranes less accumulations will occur resulting in emission of green fluorescence. CCCP is a depolarised control. Statistical analysis was done by one-way ANOVA with Bonferroni’s Multiple Comparison Test and is shown in the upperpart of the figure. ns (not significant) is P-values above 0.05. ** is P-values below or equal to 0.01. *** is P-values below or equal to 0.001.

Since the analysis of the muropeptide composition did not reveal any changes, we investigated the membrane. To evaluate effects on the bacterial membrane, the membrane potential was measured when exposed to either CBD, BAC, or the combination of the two (Fig. 5). Accumulation of the fluorescent dye DiOC_2_(3) in healthy bacteria cells with intact membrane potential results in red fluorescence (high red/green ratio), whereas lower concentrations of the dye, due to membrane potential disruption, exhibit green fluorescence (low red/green ratio), as visualised for the depolarised control using CCCP. Thus, the ratio between red and green fluorescence can reveal the state of the membrane potential. As shown in Fig. 5, even very low concentrations of CBD at 0.1 and 0.2 µg/mL as well as concentration of BAC at 16 µg/mL resulted in a significant lower red/green fluorescence ratio compared to either the untreated or the EtOH control indicating disruption of the membrane potential. However, combining BAC with CBD at either 0.1 or 0.2 µg/mL did not show any significant further membrane depolarisation compared to either CBD or BAC alone.

### Transcriptional expression analysis by qPCR

**Figure 6:**
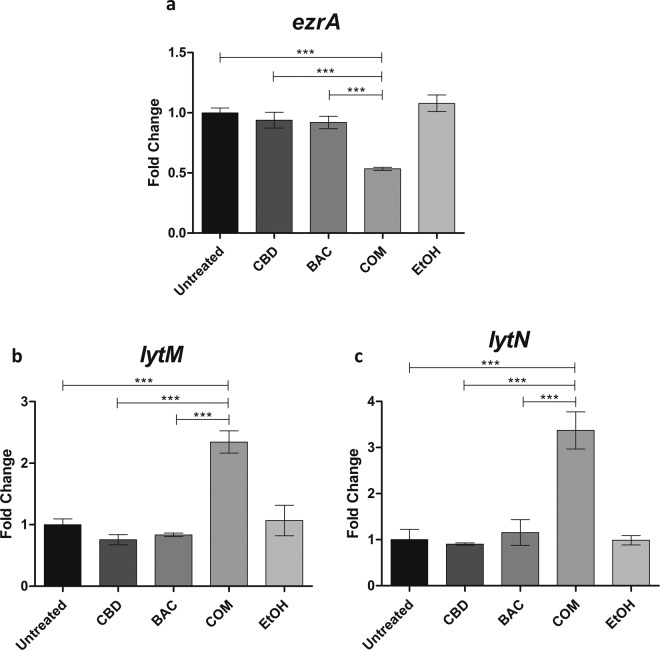
qPCR data of the divisome gene *ezrA* and autolysis genes *lytM* and *lytN* studied upon 2.5 hours treatment with either CBD, BAC, combination, EtOH or left untreated in USA300. Data was obtained using the Roche LightCycler 480 Instrument as described in Methods. Experiments were performed in four biological replicates and Cp values were generated in technical replicates. Statistical analysis was done by one-way ANOVA with Bonferroni’s Multiple Comparison Test. *** is P-values below or equal to 0.001.

With the shown defects in cell division and septum formation observed by TEM as well as decreased autolysis, we wished to identify whether expression of specific genes encoding proteins important for cell division and formation of the divisome as well as autolysis were affected by CBD and BAC. Similar to the TEM experiment, *S. aureus* USA300 was grown for 2.5 hours after exposure to CBD and/or BAC in the exponential growth phase. Analysis of transcriptional changes of selected genes (see Supplementary Table S1) involved in the divisome, cell division and autolysis of *S. aureus* upon treatment was performed by Reverse Transcriptase qPCR. Regarding the divisome and cell division genes, *ezrA* was shown to be the most regulated gene upon combination treatment at approximately 2-fold down-regulation (Fig. 6a). EzrA is an important multifunctional component of the bacterial cell divisome implicated in peptidoglycan synthesis and assembly of the cell division apparatus^19^. The results for the remaining genes analysed can be seen in Supplementary Figure S8. These data support the TEM images by showing that CBD in combination with BAC disrupts the cell division. As autolysis was shown to be decreased upon treatment with the combination of CBD and BAC, we studied the expression of selected autolysis genes. Of the genes studied, the expression of *lytM* and *lytN* showed to be highly upregulated upon combination treatment at approximately 2.5 (Fig. 6b) and 3.5 (Fig. 6c), respectively, whereas the combination treatment did not seem to affect the expression of the other genes autolysis genes (*atl*, *sle1*, *lytA*)) compared to treatment with either CBD or BAC alone (see Supplementary Information Figure S8).

## Discussion

The limited availability of effective therapies against *S. aureus* has increased the pursuit to discover new treatment strategies. Development of new antibiotics is currently undergoing an innovation gap, while research in the use of helper compound in combination with antibiotics is becoming more intense. It has previously been reviewed that many natural compounds such as flavonoids and compounds from manuka honey and teas have been reported to potentiate antibiotics^20–22^. In this study, we found that the antibacterial effects of BAC against *S. aureus* as well as other Gram-positive bacteria can be enhanced by cannabidiol originating from the Cannabis plant. The potentiation was confirmed through MIC determinations, standard growth experiments, fractional inhibitory concentration determination and time-kill assays. As expected, the combination turned out to be ineffective in Gram-negative bacteria, as BAC is a mixture of related cyclic peptides which interrupt cell wall synthesis in Gram-positive bacteria and is probably unable to cross the outer membrane in Gram-negative bacteria. BAC interferes with the dephosphorylation of bactoprenol (C_55_-isoprenyl pyrophosphate); a membrane lipid-carrier that transports peptidoglycan-precursors across the membrane for peptidoglycan biosynthesis^23,24^. The use of BAC in combination with other compounds against *S. aureus* has been studied before such as in combination with colistin^25^ and alkyl gallates^26,27^. Colistin is believed to damage the cell membrane thus increasing entry of BAC into the cell or by increasing availability of divalent ions such as Zn^2+^. This is important for the functionality of BAC^25^, whereas the mechanism underlying the alkyl gallate mediated potentiation of bacitracin is unknown. However, it has been shown to bind the bacterial membrane affecting the membrane integrity suggesting similar mechanism for synergy as suggested for colistin^28^.

The use of CBD and other cannabinoids as antibacterial agents was first described in 1976 by Van Klingeren and Ten Ham^13^ and again in 2008 by Appendino and colleagues^14^, however, since then very little has been published on this topic. CBD is a quite effective antimicrobial compound with a MIC value of 4 µg/mL against *S. aureus* USA300 and other Gram-positive bacteria. Appendino and colleagues^14^ found MIC values of CBD extracted from powdered plant material in the 0.5-1 µg/mL range towards various drug-resistant strains of *S. aureus*. However, the mechanism or how CBD and other cannabinoids affects the bacteria has not been studied so far. Endogenous endocannabinoids as anandamide (AEA) and endocannabinoid-like arachidonoyl serine (AraS) has been shown to contain poor antimicrobial properties but have a pronounced dose-dependent inhibitory effects on biofilm formation of all tested MRSA strains^12^. In this study, we have shown that the cannabinoid CBD is able to potentiate the antibacterial properties of the cell wall targeting BAC. Nevertheless, unlike in the case of the endocannabinoids, we did not find any effect on biofilm formation and breakdown in our experimental setup (Supplementary Figure S9). This may indicate a different mechanism of action for CBD.

Cell imaging is an approach to obtain indications of the mechanism or site of action of an antimicrobial compound. Cells grown in the presence of either CBD or BAC did not reveal any phenotypical changes compared to the untreated or the EtOH control. However, treatment with the combination of CBD and BAC revealed a remarkable phenotype visualised by transmission electron microscopy. The TEM images showed bacteria with several septa causing lack of cell separation during cell cycle and a distorted cell membrane. The lack of cell separation and termination of the cell cycle is consistent with the Triton X-100 induced autolysis assay showing a decrease in autolysis upon combination treatment. We therefore thought to study the expression of genes encoding proteins involved in the autolysis and found the expression of *lytM* and *lytN* to be upregulated upon combination treatment. In a study of gene silencing of the major regulator of cell wall metabolism *walRK*, *S. aureus* was shown to have similar phenotype as observed in this study; several septa and initiation of septa^29^. In addition, they found that by increasing the expression of *lytM*, they could restore the viability of the cells even though the cells had still formed several septa. Based on this, the increased expression of *lytM*, and perhaps also *lytN*, might be due to a kind of self-defense mechanism trying to restore the cell viability. Regarding the several septa formation, similar characteristics have been recorded by others as well, e.g. by treating *S. aureus* with the wall teichoic acid biosynthesis inhibitor targocil ^30,31^, causing both decreased autolysis and impaired cell division as visualised by several septa formations. In addition, formation of several septa has been visualised by others by creation of gene knockouts or by performing gene silencing of genes important for cell cycle regulation. Pang and colleagues showed this phenotype in a Δ*noc* strain, lacking a very important cell division regulator^32^. In addition, Stamsas and colleagues found effects on septum formation in a Δ*cozEa* strain upon gene silencing of *cozEb*, encoding proteins which together are important for proper cell division in *S. aureus* and which interacts with the major cell division protein EzrA^33^. Furthermore, construction of a conditional *ezrA* mutant has also shown to cause impaired cell division and several septa formations in *S. aureus*^34^. The fact that *ezrA* is downregulated upon exposure to the combination of CBD and BAC by approximately two-fold, indicates that the combination of CBD and BAC affects the cell division in *S. aureus*. Steele and colleagues^19^ showed that *S. aureus* cells partially depleted of EzrA cannot divide without sufficient levels of EzrA. The authors also showed that EzrA is required for peptidoglycan synthesis. Nevertheless, the combination of CBD and BAC in our experimental setup does not seem to have a noteworthy effect on peptidoglycan synthesis, or at least not on the composition of the peptidoglycan nor the degree of cross-linking, as the muropeptide analysis showed a similar pattern in the chromatograms of the untreated cells as for the CBD and BAC treated cells. However, whether the decreased expression of *ezrA* is a direct or a secondary effect of the combination treatment is unknown and will be studied further in the future.

The exact mechanism of CBD potentiation of BAC is not yet fully understood; however, it was visualised that the combination did cause cell division complications and envelope irregularities. As mentioned above regarding the combination of colistin and BAC and presumably alkyl gallates, one would argue that CBD could have similar mechanism, that is affecting the membrane as visualised by the membrane potential disruption. This causes either increase of BAC entry into the cell or increased divalent ion availability for BAC. On the other hand, the mechanism of potentiation seems to be specific for bacitracin, since no particular synergy was observed when combining CBD with either dicloxacillin, daptomycin, nisin or tetracycline indicating other mechanisms for CBD mediated potentiation of BAC than an increased uptake due to disrupted membrane.

## Conclusion

In this study, we present a putative novel antimicrobial combination for treatment of Gram-positive bacterial infections using the cannabinoid cannabidiol and the cell wall targeting antibiotic bacitracin.

Through growth experiments, it was interestingly found that CBD was able to potentiate the effects of BAC against *S. aureus* USA300 and other Gram-positive bacteria. However, it was found to be ineffective against Gram-negative bacteria. Upon treatment with the combination of CBD and BAC, it was revealed by TEM that the morphology of the cells had changed compared to cells treated with either CBD or BAC alone or left untreated. The cells showed several septa formations indicating lack of cell separation during cell division causing reduced autolysis, as well as an irregular membrane. In addition to this, a very important cell division gene, *ezrA*, turned out to be transcriptionally down regulated upon combination treatment. Changes observed in morphology was not caused by compositional changes in the cell wall muropeptide composition. Membrane potential changes for the combination of CBD and BAC compared to either CBD or BAC treatment alone did not reveal the mechanism of action for the combination of CBD and BAC. Future studies are therefore focused on the cell division and cell envelope to identify the mechanism of action.

## Methods

### Bacteria and growth conditions

The resistant *Staphylococcus aureus* strain, MRSA USA300 FPR3757^35^, was the main bacterium used throughout this study. MRSA was grown in Brain Heart Infusion (BHI) or Müeller Hinton (MH) media on plate or in liquid cultures with agitation at 37 °C. Additional bacteria *Enterococcus faecalis* (13-327129), Methicillin-resistant *Staphylococcus epidermidis* (933010 3F-16 b4), *Listeria monocytogenes* EGD, *Pseudomonas aeruginosa* (PA01), *Salmonella typhimurium* (14028), *Klebsiella pneumoniae* (CAS55), and *Escherichia coli* (UTI89) were either grown in BHI, MH or Lysogeny Broth (LB) media on plate or in liquid cultures with agitation at 37 °C. As CBD (Sigma Aldrich) was dissolved in EtOH, a control using same volume of 96% EtOH was constructed.

### Minimum inhibitory concentration (MIC) and Fractional Inhibitory concentration (FIC)

The MIC was determined using the broth microdilution method^36^. The MIC was interpreted as the lowest concentration at which no growth was observed. Briefly, MIC measurements were performed using the MH or BHI medium in 96-well plates (Nunc A/S or Sarstedt). A total volume of 100 μl with a bacterial inoculum of approximately 5 × 10^5^ CFU/mL was incubated with two-fold dilution series of the compound or antibiotic of interest for the MIC determination and incubated at 37 °C for 16–22 hours with agitation. For the FIC index determination, ¼ volume of each compound or liquid media was added to the wells in the 96-well plate and final ½ volume with same bacterial inoculum was added to the wells afterwards. The plate was incubated as mentioned above. The MIC and FIC determination were performed using at least three biological replicates. Growth was determined using a Synergy H1 Plate Reader (BioTek). The FIC index was calculated using this formula:

$${FIC}\,{Index}=\frac{{MI}{{C}}_{{A}}\,{in}\,{Combination}}{{MI}{{C}}_{{A}}}+\frac{{MI}{{C}}_{{B}}\,{in}\,{Combination}}{{MI}{{C}}_{{B}}}$$

and can be used to determine presence of synergy between two compounds. MIC_A_ and MIC_B_ in combination indicate the MIC value of compound A or B, when combined with the other compound (A or B). The FIC index were defined as follow: FICI ≤ 0.5 shows synergy, FICI 0.5-4 shows indifference, and FICI > 4 shows antagonism^37^.

### Growth experiments and time-kill assay

For micro dilution growth experiments, a 96-well plate (Nunc Edge) was prepared with different concentrations of the compounds of interest in MH media. Diluted overnight cultures (OD_600_ nm of 0.005) were added to each well. The plate was incubated at 37 °C (without agitation) for 24 hours. Using an oCelloScope (BioSense Solutions ApS), the bacterial density was measured over a period of 24 hours using the UniExplorer software. Data retrieved was obtained as Background Corrected Absorption (BCA), calculated by an algorithm enabling determination of bacterial growth kinetics resulting from images taken with the oCelloScope camera^38,39^. Experiments were performed with at least three biological replicates.

For macro dilution growth experiment, an ON culture was diluted to OD_600_ 0.02 in BHI media in an Erlenmeyer Flask and placed in a water bath at 37 °C with agitation. Bacterial growth was determined by turbidity measurements at OD_600_ nm.

The time-kill assay was performed as previously described^40^. Briefly, a culture grown to OD_600_ 0.2 in BHI, was split, and treated with CBD or BAC alone and in combination. An untreated control was included. Viability was monitored by OD_600_ readings and CFU/mL determinations by spotting 10-fold serial dilutions on MH agar plates. The viability assay was performed twice with similar results.

### Transmission electron microscopy (TEM)

Cultures were prepared for TEM according to Thorsing *et al*.^40^. Briefly, USA300 was grown in BHI media at 37 °C with agitation from OD_600_ 0.02 to start exponential growth at 0.2. The culture was diluted 5 times in BHI media and split into different flasks. The cultures were either left untreated or treated with 1 µg/mL CBD and/or 16 µg/mL BAC or ethanol. The cells were incubated at 37 °C with agitation for 2 hours and 30 minutes. Treated cells were harvested and the pellet was washed twice in PBS followed by fixation ON in 2% glutaraldehyde diluted in 0.04 M phosphate buffer pH = 7.4. The fixated cells were washed in 0.1 M phosphate buffer pH = 7.4 and the pellet was resuspended in 15% bovine serum albumin and incubated at 20 °C for 1 hour and 15 minutes. The cells were centrifuged and fixed again ON in 2% glutaraldehyde at 4 °C. Fixated cell pellets were cut into pieces and washed three times using 0.1 M phosphate buffer pH 7.4, followed by staining with 1% OsO_4_ for 60 minutes at 4 °C. The samples were dehydrated using increasing concentrations of ethanol (50–99%) at 4 °C and then embedded in epon TAAB-812. The samples were cut into ultra-thin sections using an ultra-microtome and collected on a nickel grid. The sections were stained using 3% uranyl acetate for 14 minutes at 60 °C followed by a wash using water and then stained using lead citrate for 6 minutes at room temperature. Finally, the samples were washed in 20 mM NaOH and water and then dried. The sections were analysed by transmission electron microscopy using a Philips EM 208 Microscope equipped with a Quemsa TEM CCD camera and an iTEM Digital Imaging Platform software. The experiment was carried out using two biological replicates.

### Fluorescence microscopy

Cells were grown and treated as mentioned for transmission electron microscopy. After treatment, cells were washed in PBS and incubated at room temperature with 5 µg/mL Bocillin-FL for 4 minutes followed by wash in PBS. The cells were then incubated with 100 µg/mL DAPI for 4 minutes followed by PBS wash. Stained cells were added to a Poly-L-Lysine treated glass slide and visualised using Olympus IX83 fluorescence microscope with 405 nm for DAPI and 488 nm for Bocillin-FL. Images were processed using ImageJ (NIH).

### Autolysis assay

The autolysis assay was performed according to Campbell *et al*.^31^. Briefly, ON culture of *S. aureus* USA300 was diluted in BHI to OD_600_ 0.02 and grown to early exponential phase OD_600_ 0.2 at 37 °C with agitation. The culture was treated with either 1 µg/mL CBD, 16 µg/mL BAC, the combination of CBD and BAC, the solvent EtOH, or left untreated and incubated for one hour at 37 °C degrees with agitation. After incubation, cells were washed in PBS pH 7.2 and pellet was resuspended in 50 mM Tris-HCl with or without 0.05% Triton X-100 and adjusted to OD_600_ 1.0 and incubated at 30 °C with gentle agitation. Turbidity measurements were performed every 30 or 60 minutes for five hours at OD_600_. The autolysis assay was carried out in three biological replicates.

### Muropeptide isolation and analysis by reverse phase HPLC

Muropeptides were isolated according to Kühner *et al*.^18^ with minor changes. Briefly, an ON culture of *S. aureus* USA300 was treated and grown as mentioned above for TEM. After 2.5 hours treatment, the cells were harvested at 10.000x G and the pellet was resuspended in 0.1 M tris/HCl containing 0.25% SDS and heated to 100 °C for 30 minutes. To remove SDS the samples were washed at least 15 times in sterile ddH_2_O and absence of residual SDS was confirmed according to Heyashi, 1975^41^. The samples were sonicated for 30 minutes in a sonicator bath followed by DNase and RNase treatment using DNase I (15 µg/mL) and RNase A (60 µg/mL) and incubated at 37 °C for 1 hour followed by trypsin digestion (50 µg/mL) for 1 hour at 37 °C. Enzymes were inactivated at 100 °C for 3 minutes. To remove wall teichoic acids, the samples were incubated with 1 M HCl at 37 °C for 4 hours with agitation. The samples were washed to pH = 5–6 and resuspended in digestion buffer. Cell walls were digested with mutanolysin (5000 U/mL) (Sigma) at 37 °C with agitation for 17 hours. The samples were then centrifuged, and supernatant was moved to a fresh tube followed by addition of 50 µL reduction solution containing 10 mg/mL NaBH_4_ and left at room temperature for 20 minutes with lids open to reduce MurNac. The reaction was stopped using 15 µL of 85% phosphoric acid.

Separation of the samples were carried out with a flow rate of 250 µL/min using an Agilent 1260 Infinity RP-HPLC (Agilent Technologies) and an Xselect Peptide CSH C18, 130 A, 3.5 µm, 2.1 mm ×150 mm column (Waters) heated to 52 °C. The peptides were eluted by a gradient of solvent B (0.06% trifluoroacetic acid (TFA)/35% methanol) and solvent A (0.06% TFA) as described^18^. The muropeptide isolation and subsequent separation was carried out in three biological replicates.

Cross-linking was calculated as -described in^40^:

$${Cross}{ \mbox{-} }{Linking}\, \% =0.5\,{x}\,{dimer}\,( \% )+0.67\,{x}\,{trimer}( \% )+0.9\,{x}\,{oligmer}\,( \% )$$

### Membrane potential

The bacterial membrane potential was analysed using BacLight Bacterial Membrane Potential Kit (ThermoFisher) and the experiment was performed according to the manufacturer’s recommendations. Briefly, an ON cultures of USA300 was diluted to OD_600_ 0.02 and grown to 0.3 in BHI media at 37 °C with agitation. The culture was diluted 1:100 in PBS and split into separate tubes. The samples including an ethanol control were either left untreated or treated with either 5 µM CCCP (depolarised control), 0.1 or 0.2 µg/mL CBD and/or 16 µg/mL BAC and incubated at room temperature for 5 minutes. Following the incubation, the dye diOC_2_(3) was added to a final concentration of 0.03 mM in each sample except for an unstained control and left to stain for at least 15 minutes at room temperature protected from light. The samples were analysed using BD FACSAria II flow cytometer and FACSDiva Version 6.1.2 software. For each sample, 10^4^ events were analysed using a laser emitting at 488 nm and fluorescence was collected in the red and green channels. The experiment was carried out in three biological replicates.

### Isolation of total cellular RNA, DNase treatment and cDNA synthesis

Cultures of *S. aureus* USA300 were prepared, diluted and treated as described for TEM. After 2.5 hours of treatment, samples were harvested for RNA purification. RNA was purified by a hot acid-phenol procedure^42^ using FastPrep and FastPrep beads and treated with 0.2 units of DNase I (NEB) for 15 minutes at 37 °C followed by heat inactivation. cDNA was synthesised using the High-Capacity cDNA Reverse Transcriptase Kit (ThermoFisher Scientific) and was performed according to the manufacturer’s recommendations. Briefly, the cDNA synthesis was carried out at 25 °C for 10 minutes followed by 37 °C for 45 minutes and finally the enzyme was inactivated at 85 °C for 5 minutes. A No Template Control (NTC) and a No Reverse Transcriptase Control (NRT) was created as well. The experiment was performed using four biological replicates.

### Quantitative polymerase chain reaction

Reverse Transcriptase qPCR was performed using the Roche LightCycler 480 Instrument. For each reaction, 5 µL RealQ Plus Master Mix Green 2×(Ampliqon), 0.75 µL 10 µM primers (Supplementary Table S1), 1 µL sterile ddH_2_O and 2.5 µL sample were used. Each reaction was made in technical duplicates. Pre-incubation was set at 95 °C for 15 minutes, the amplification cycle was set at 95 °C for 15 seconds followed by 60 °C for 45 seconds and then 72 °C for 45 seconds for 45 cycles. A melting curve was created in the end of the procedure by heating to 95 °C for 5 seconds 60 °C for 20 seconds and then 97 °C continuously. Data was retrieved using LightCycler 480 Software version 1.5.1.62. Data were normalised using *gyrB* as a reference gene.

### Statistical analysis

P-values were calculated by a one-way ANOVA with Bonferroni’s Multiple Comparison Test for qPCR and membrane potential data. A 2-way ANOVA with Bonferroni’s Multiple Comparison Test was used for the autolysis assay. Significance was determined based on the P-values visualised in the figures. ns (not significant) is P-values above 0.05. * is P-values below or equal to 0.05. ** is P-values below or equal to 0.01. *** is P-values below or equal to 0.001.

## Supplementary information

Supplementary Data.
