# Supplementary material for: Cannabidiol is an effective helper compound in combination with bacitracin to kill Gram-positive bacteria
Source: Sci Rep. 2020 Mar 5;10:4112. doi: 10.1038/s41598-020-60952-0 (PMC7057955; doi:10.1038/s41598-020-60952-0)
Supplement: Supplementary file 1 — Supplementary Data. [file 41598_2020_60952_MOESM1_ESM.pdf]

Cannabidiol is an effective helper compound in combination with bacitracin to kill Gram-positive bacteria

Claes Søndergaard Wassmann[1], Peter Højrup[2], and Janne Kudsk Klitgaard[1,3*]

[1] Department of Biochemistry and Molecular Biology, Research Unit of Molecular Microbiology, University of Southern Denmark, Odense, Denmark

[2] Department of Biochemistry and Molecular Biology, Protein Research Group, University of Southern Denmark, Odense, Denmark

[3] Institute of Clinical Research, Research Unit of Clinical Microbiology, University of Southern Denmark, Odense, Denmark.

* Corresponding author. Mailing address: Department of Biochemistry and Molecular Biology, University of Southern Denmark, Campusvej 55, DK-5230 Odense M, Denmark. Phone: +45 6550 2376. Fax: +45 6550 2467. E-mail address: jkklitgaard@health.sdu.dk

Supplementary Table S1

Overview of primers used in the study

Primers	Sequence (5’-3’)
qPCR_ftsZ_F	AAAGCTGCAGAGGAATCTCG
qPCR_ftsZ_R	TTTAGCAACGACTGGTGCTG
qPCR_ezrA_F	ATTTGGTGAGGCAGCAAGTC
qPCR_ezrA_R	GCTATATGGTTGTGCGCTTG
qPCR_ftsW_F	CTTGTGTGTGTTGGGATTGC
qPCR_ftsW_R	TTGATGATCCACCAAAGCTG
qPCR_ftsH_F	GATTGATGCTGTTGGTCGTC
qPCR_ftsH_R	CCGAAACCATCCATTTCAAC
qPCR_ftsA_F	GGCGAGAAATTTCACAATGG
qPCR_ftsA_R	TGTGTCTTTGATTGCTTGTCG
qPCR_ftsL_F	GATACGCGAGGAAAGATTGC
qPCR_ftsL_R	ATCGTTCTCAAGGCTCATCC
qPCR_divIB_F	CGACTCGATTGATGAGGAAAC
qPCR_divIB_R	TTGTCGCTTACGTCTTAACTTCC
qPCR_divIC_F	ATGCGTGTTGTTCGTAGGC
qPCR_divIC_R	CGCTCCTGTGCATCAATATC
qPCR_divIVA_F	GATCGCGTTTCCGTATGTTAG
qPCR_divIVA_R	TCAAGCGTCACTTGTTCAGC
qPCR_sepF_F	GCGGTACTGTTTATGCAATCG
qPCR_sepF_R	TGGTCTGTAATGCTTCCAGCTAC
qPCR_atl_F	GACCCTGCTATTGTCCAACC
qPCR_atl_R	CGCTGATTGATTAGCACGAG

qPCR_lytM_F	TCAGCAAGTAAAGCGACAGC
qPCR_lytM_R	TTTCAGGCATTGCATAGTCG
qPCR_sle1_F	TCAGGATCTGCAACAACGAC
qPCR_sle1_R	CCTTTACCAATTTCAGCACGAC
qPCR_lytA_F	TGGTGGTGCAAAGTTCATTC
qPCR_lytA_R	TATCTGCCCAGCGAATGTC
qPCR_lytN_F	TGCCAATGACACCATTAGTAGAAC
qPCR_lytN_R	ACCGTCGAAATCCCATCC
gyrB_fwd3	GAAGCATTAGCTGGTTATGCAA
gyrB_rev3	CCACGTCCGTTATCCGTTAC

Supplementary Figure S1

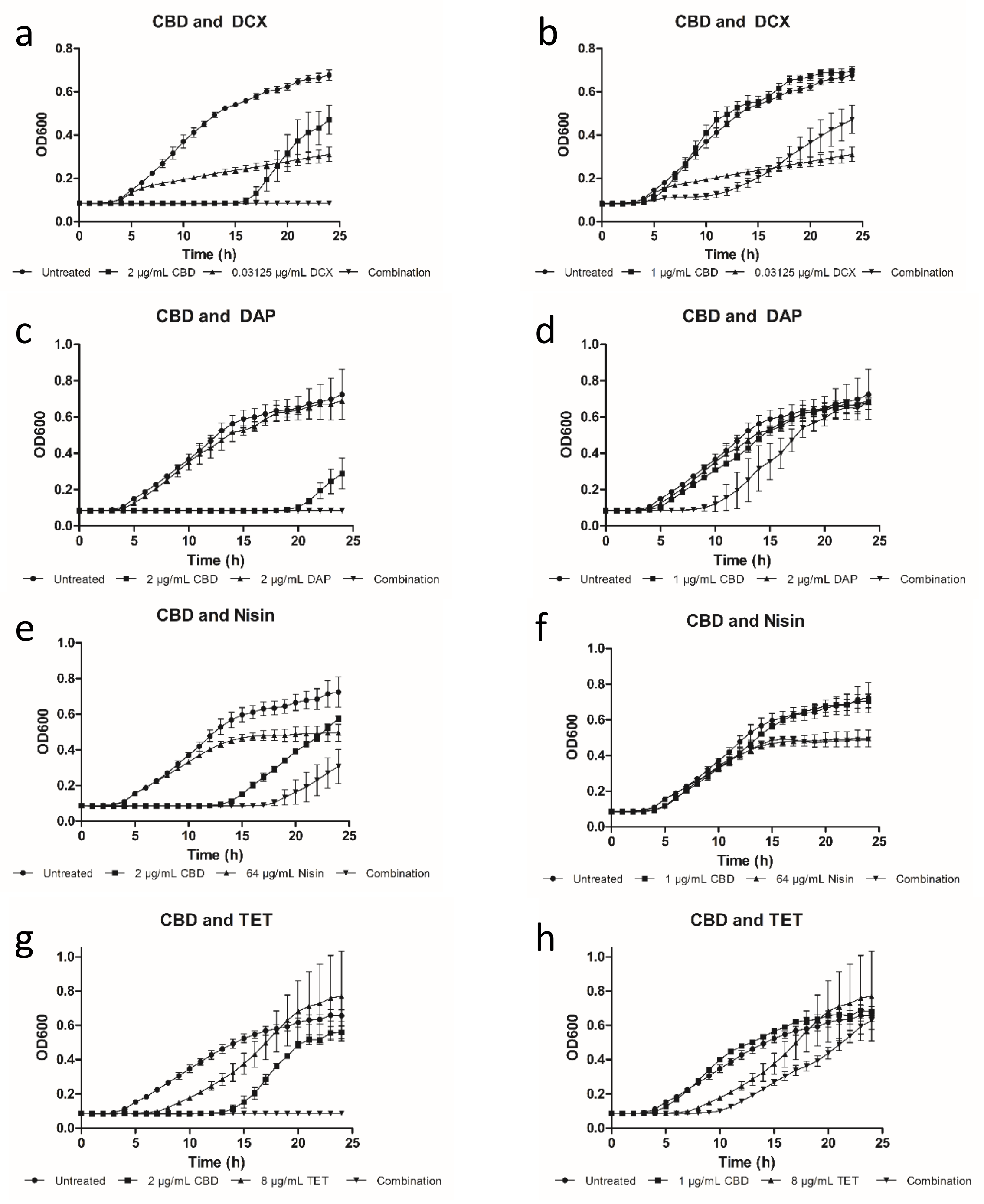

Figure S1: Growth experiment of USA300 treated with CBD in combination with various antibiotics a-b) Dicloxacillin (DCX), c-d) Daptomycin (DAP), e-f) Nisin and g-h) Tetracycline (TET).
The 96-well plate was prepared with antibiotics and media as mentioned in Methods. ON cultures were diluted to OD600 0.001 and added to the 96-well plate. Growth (OD600) was measured every hour using a Synergy H1 Plate Reader (Biotek) for 24 hours at 37 degrees with 15 seconds agitation before each measurement. The experiment was performed in three biological replicates.

Supplementary Figure S2

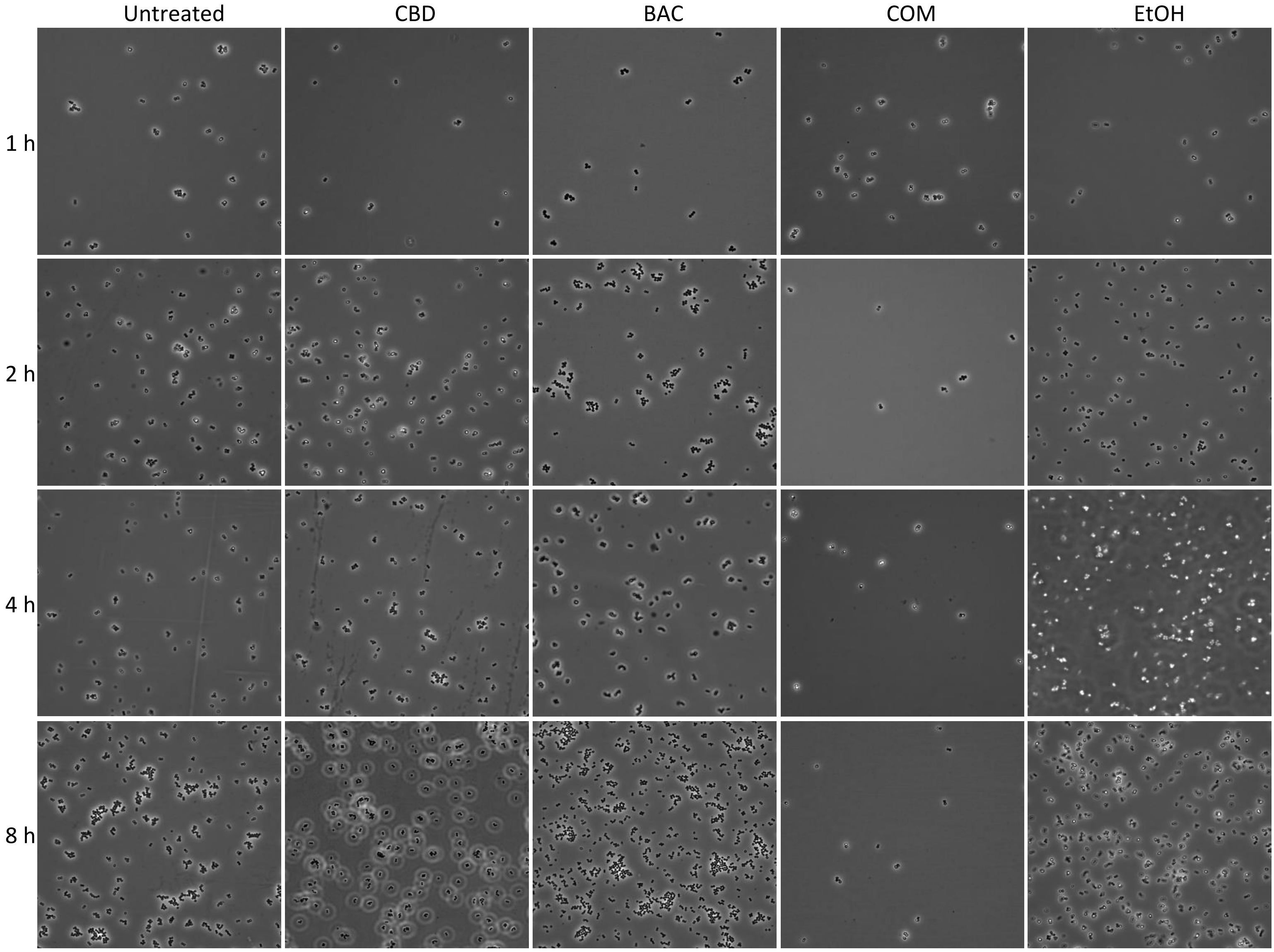

Figure S2: Microscopy of *S. aureus* USA300 left untreated, treated with 1.5 µg/mL CBD, 20 µg/mL BAC, combination of CBD and BAC or EtOH at 1, 2, 4 and 8 hours post treatment.

Supplementary Figure S3

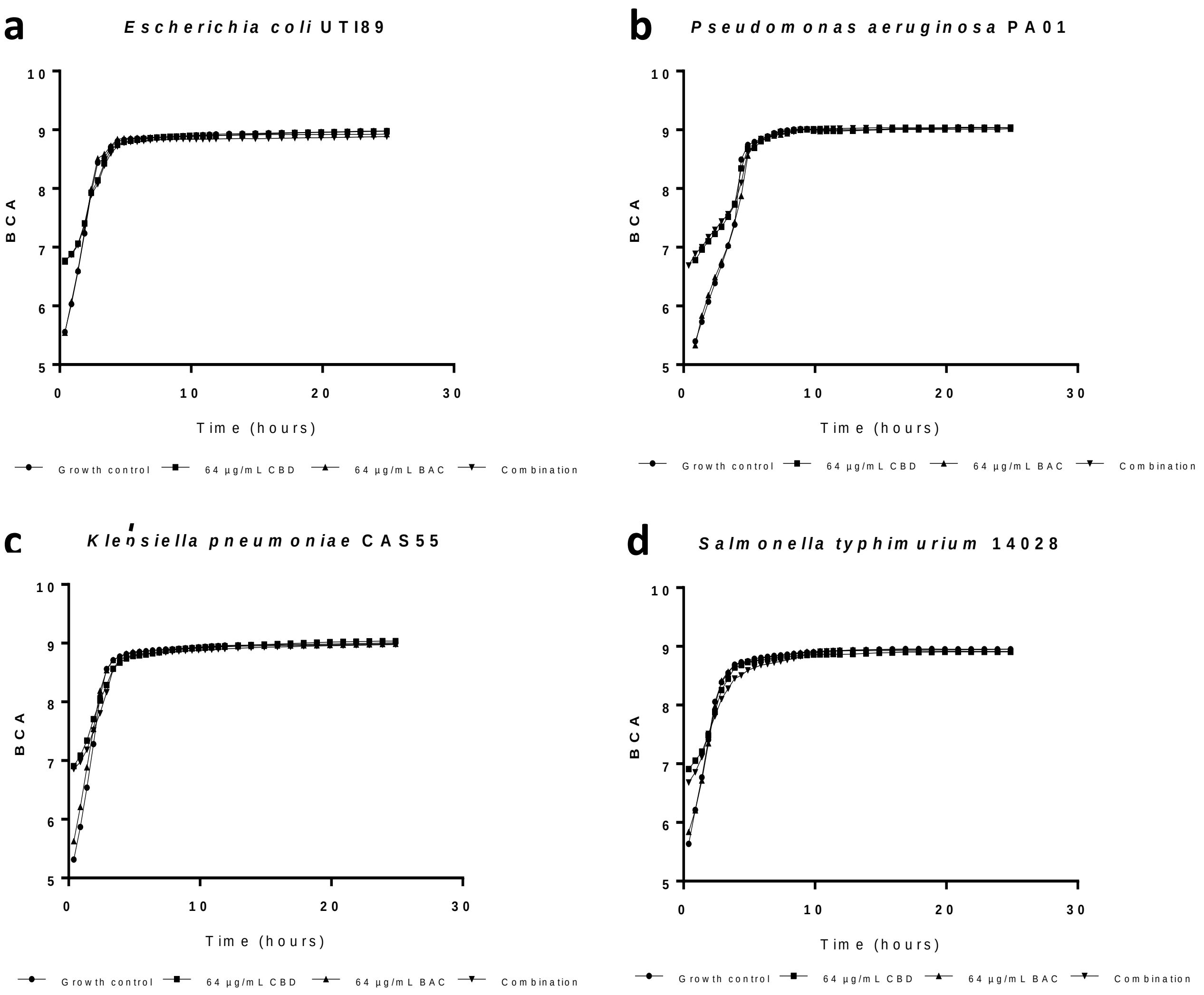

Figure S3: Growth curves of cannabidiol (CBD) in combination with bacitracin (BAC). Bacterial density (BCA: Background corrected absorption) was measured using an oCelloScope for 24 hours; a) *Escherichia coli* UTI89, b) *Pseudomonas aeruginosa* PA01, c) *Klebsiella pneumonia* CAS55, d) *Salmonella thyphimurium* 14028.

Supplementary Figure S4

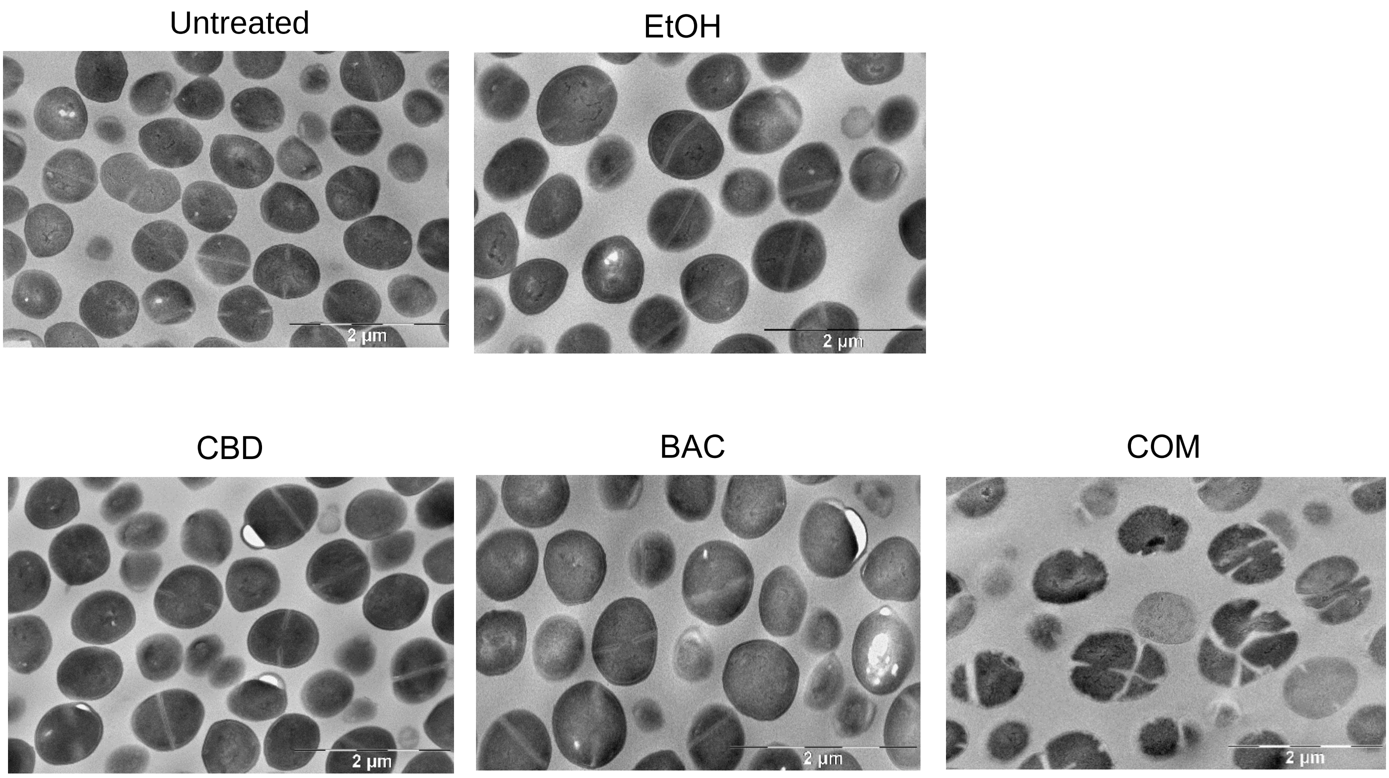

Figure S4: Overview images of morphology of USA300 FPR3757 following untreated, treatment with EtOH, treatment with cannabidiol (CBD) and/or bacitracin (BAC). Cultures were subjected to the drugs for 2.5 hours as described in Methods. Morphology was imaged by transmission electron microscopy.

Supplementary Figure S5

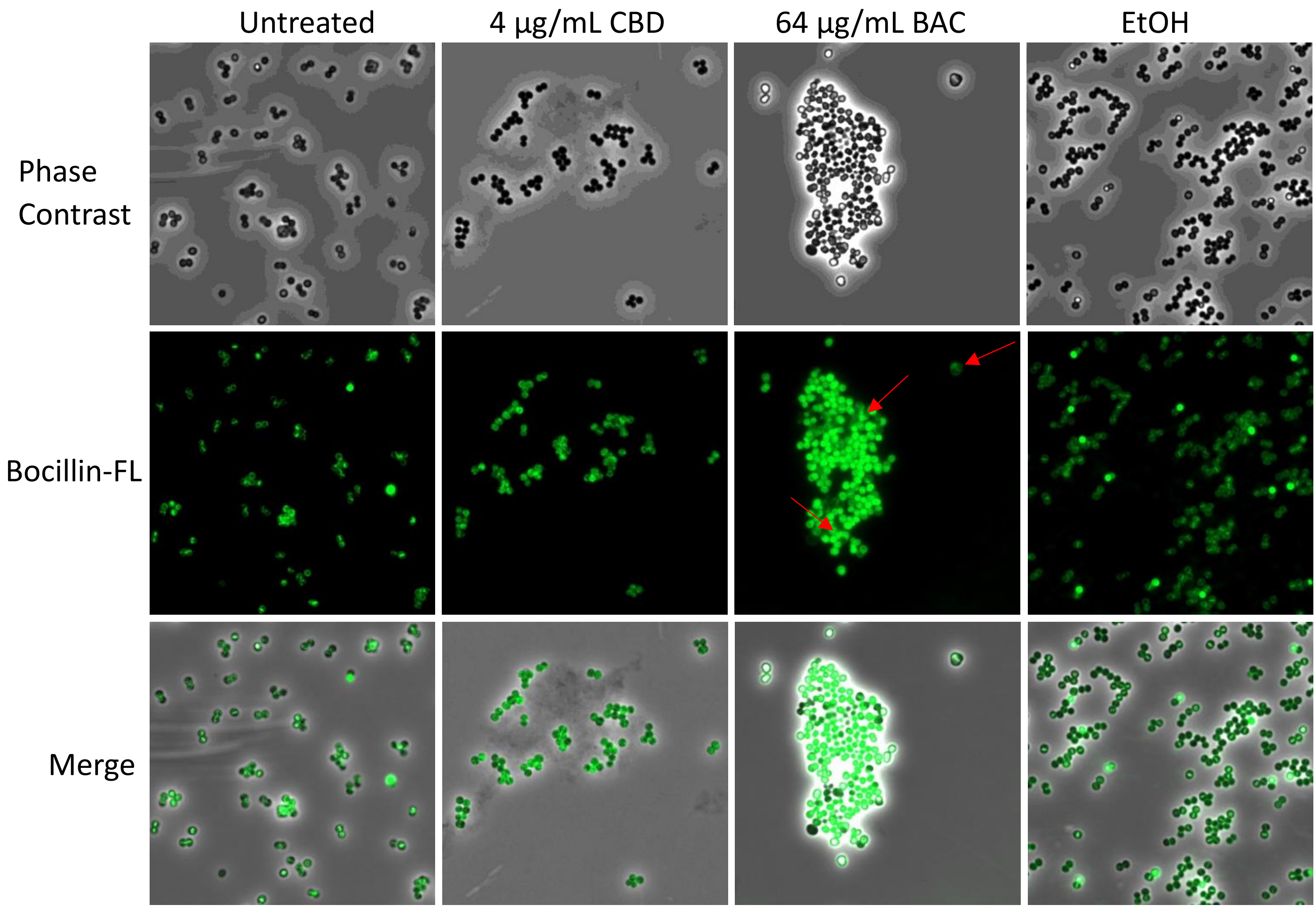

Figure S5: Microscopy of USA300 left untreated, treated with 4µg/mL CBD, 64µg/mL BAC or EtOH. Red arrows points towards cells with multiple septa.

Supplementary Figure S6

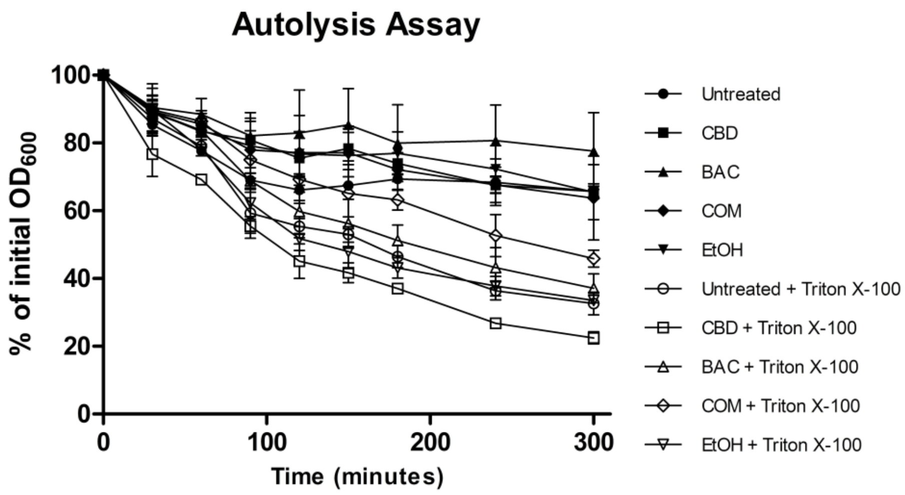

Figure S6: Effects of cannabidiol (CBD) and bacitracin (BAC) on autolysis. Unstimulated and Triton X-100 stimulated autolysis of USA300 grown in BHI to early exponential phase.

Supplementary Table S2

Timepoint	Untreated vs COM		CBD vs COM		BAC vs. COM	
**0**	P > 0.05	ns	P > 0.05	ns	P > 0.05	ns
**30**	P > 0.05	ns	P < 0.05	*	P > 0.05	ns
**60**	P > 0.05	ns	P < 0.01	**	P > 0.05	ns
**90**	P < 0.01	**	P < 0.001	***	P > 0.05	ns
**120**	P < 0.05	*	P < 0.001	***	P > 0.05	ns
**150**	P > 0.05	ns	P < 0.001	***	P > 0.05	ns
**180**	P < 0.01	**	P < 0.001	***	P > 0.05	ns
**240**	P < 0.01	**	P < 0.001	***	P > 0.05	ns
**300**	P < 0.05	*	P < 0.001	***	P > 0.05	ns

Statistical analysis by 2-way ANOVA with Bonferroni's Multiple Comparison Test on Triton X-100 stimulated samples. ns (not significant) P>0.05, * P≤0.05, ** P≤0.01, *** P≤0.001

Supplementary Figure S7

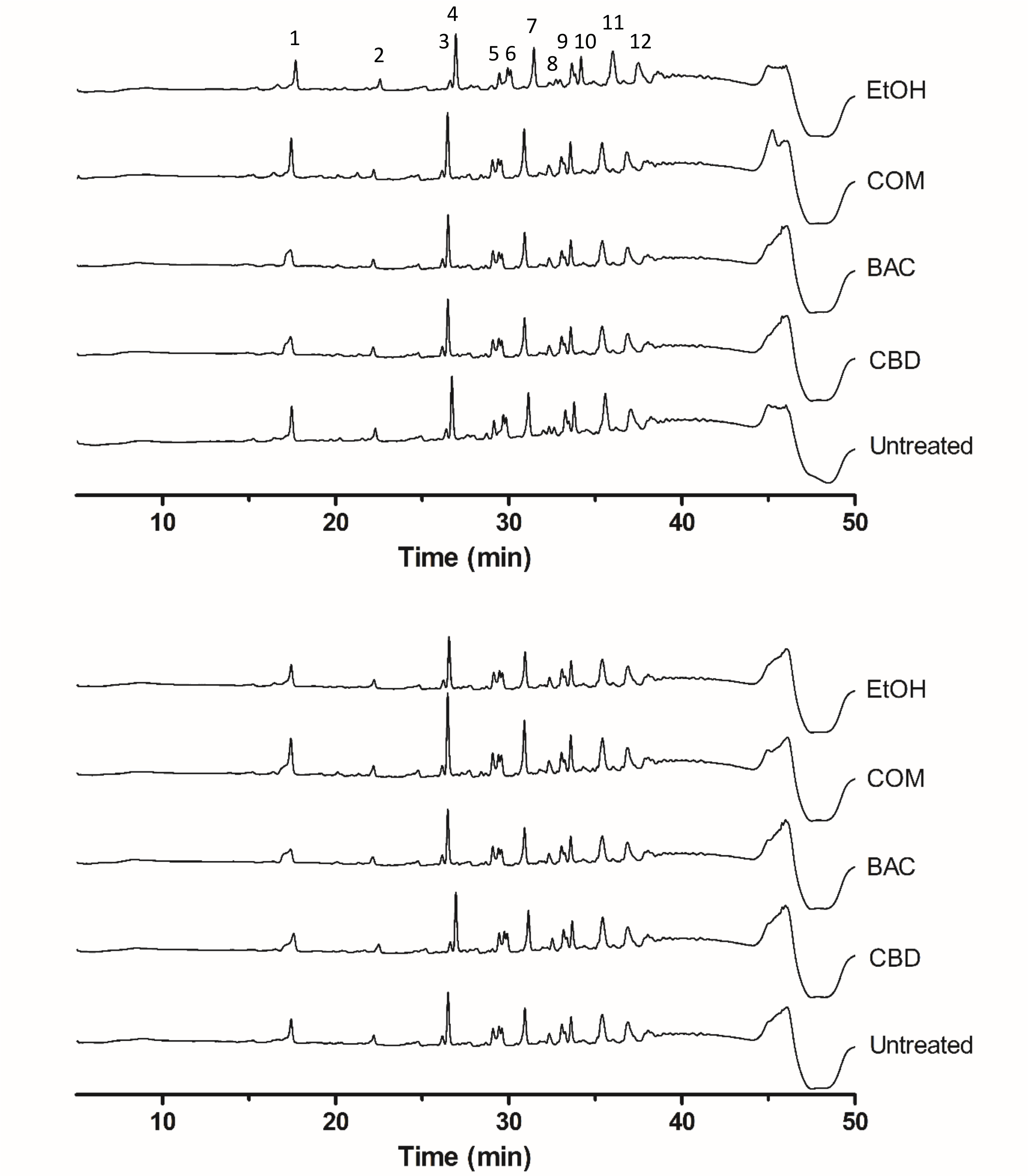

Figure S7: Two biological replicates showing the effect of CBD and BAC on the muropeptide composition of USA300 peptidoglycan. Peptidoglycan was isolated from cultures grown to exponential phase in the absence or presence of CBD or BAC and muropeptide compositions were analysed by HPLC as described in Methods. Muropeptide analysis was performed in three biological replicates with similar profiles. X-axis show retention time in minutes.

Supplementary Table S3

Peak #	GC mean	GC s.d.	CBD mean	CBD s.d.	BAC mean	BAC s.d.	COM mean	COM s.d.	EtOH Mean	EtOH s.d.
1	11.25	0.90	12.16	0.31	12.35	0.30	12.72	1.72	9.95	0.77
2	3.86	0.16	3.5	0.30	4.01	0.25	2.91	0.30	3.63	0.51
3	2.66	0.12	2.62	0.06	2.83	0.04	2.20	0.07	2.68	0.14
4	12.88	0.38	13.58	0.44	14.14	0.13	15.78	0.54	13.19	0.12
5	4.08	0.93	3.93	0.89	3.85	0.05	4.01	0.93	4.07	0.93
6	9.34	0.48	7.19	1.26	6.05	0.08	7.02	0.99	9.41	0.95
7	11.17	0.39	12.09	0.44	12.45	0.17	13.90	0.80	11.52	0.29
8	3.32	0.42	3.05	0.10	3.28	0.01	3.49	0.12	3.61	0.43
9	7.67	0.46	8.03	0.31	7.83	0.14	6.90	0.70	7.86	0.21
10	5.91	0.36	6.51	0.32	6.97	0.13	7.30	0.35	6.19	0.21
11	15.28	1.18	14.94	0.63	14.17	0.69	13.19	1.20	15.04	1.02
12	12.56	0.27	12.45	0.23	12.06	0.45	10.58	0.58	12.85	0.59

Mean value and standard deviation of the relative quantification of the muropeptide fractions.

Supplementary Table S4

Peak #	GC vs CBD	GC vs BAC	GC vs COM	GC vs EtOH	CBD vs BAC	CBD vs COM	CBD vs EtOH	BAC vs COM	BAC vs EtOH	COM vs EtOH
#1	ns	ns	ns	ns	ns	ns	**	ns	**	***
#2	ns	ns	ns	ns	ns	ns	ns	ns	ns	ns
#3	ns	ns	ns	ns	ns	ns	ns	ns	ns	ns
#4	ns	ns	***	ns	ns	**	ns	ns	ns	***
#5	ns	ns	ns	ns	ns	ns	ns	ns	ns	ns
#6	**	***	**	ns	ns	ns	**	ns	***	**
#7	ns	ns	***	ns	ns	*	ns	ns	ns	**
#8	ns	ns	ns	ns	ns	ns	ns	ns	ns	ns
#9	ns	ns	ns	ns	ns	ns	ns	ns	ns	ns
#10	ns	ns	ns	ns	ns	ns	ns	ns	ns	ns
#11	ns	ns	**	ns	ns	*	ns	ns	ns	*
#12	ns	ns	*	ns	ns	*	ns	ns	ns	**

Statistical analysis by 2-way ANOVA with Bonferroni's Multiple Comparison Test on the muropeptide fractions for the various treatments. ns (not significant) P>0.05, * P≤0.05, ** P≤0.01, *** P≤0.001.

Supplementary Table S5

Fraction size	GC	CBD	BAC	COM	EtOH
Monomer	15.12	15.62	16.36	15.63	13.57
Dimer	28.97	27.32	26.87	29.01	29.36
Trimer	14.49	15.14	15.73	17.39	15.13
Oligomer	41.43	41.93	41.03	37.98	41.94
% Cross-Linking	61.48	61.53	60.91	60.33	62.56

Mean values of the relative quantification of the muropeptide fraction sizes. Monomer (peak #1-2), dimer (peak #3-6), trimer (peak #7-8) and oligomer (#9-12).

Supplementary Figure S8

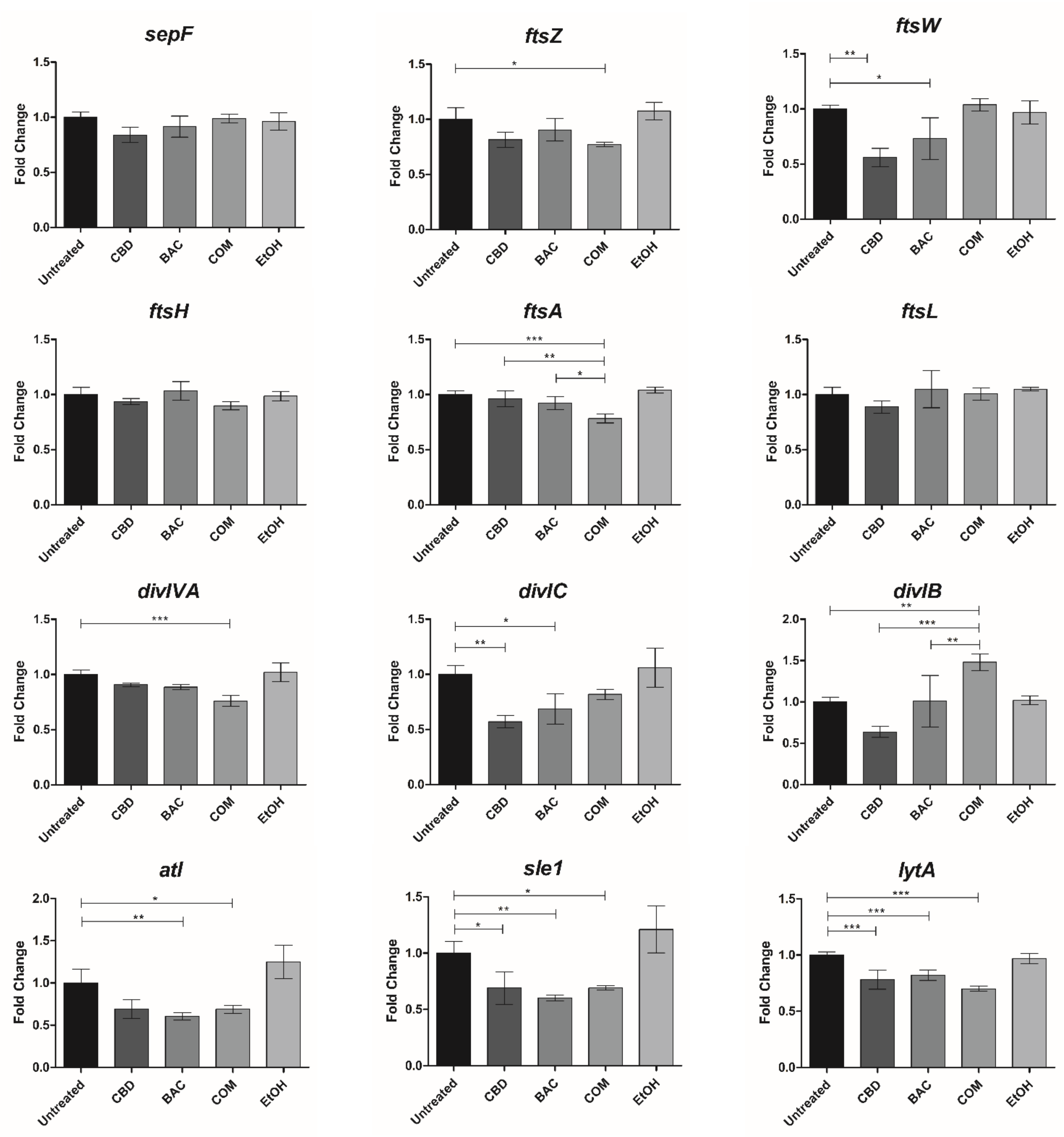

Figure S8: qPCR data of the cell division and autolysis related genes studied upon 2.5 hours treatment with either CBD, bacitracin or combination in USA300. Data was obtained using the Roche LightCycler 480 Instrument as described in Methods. Experiments were performed in four biological replicates and Cp values were generated in technical replicates. Statistical analysis was done by one-way ANOVA with Bonferroni's Multiple Comparison Test. * is P-values below or equal to 0.05. ** is P-values below or equal to 0.01. *** is P-values below or equal to 0.001.

Supplementary Figure S9

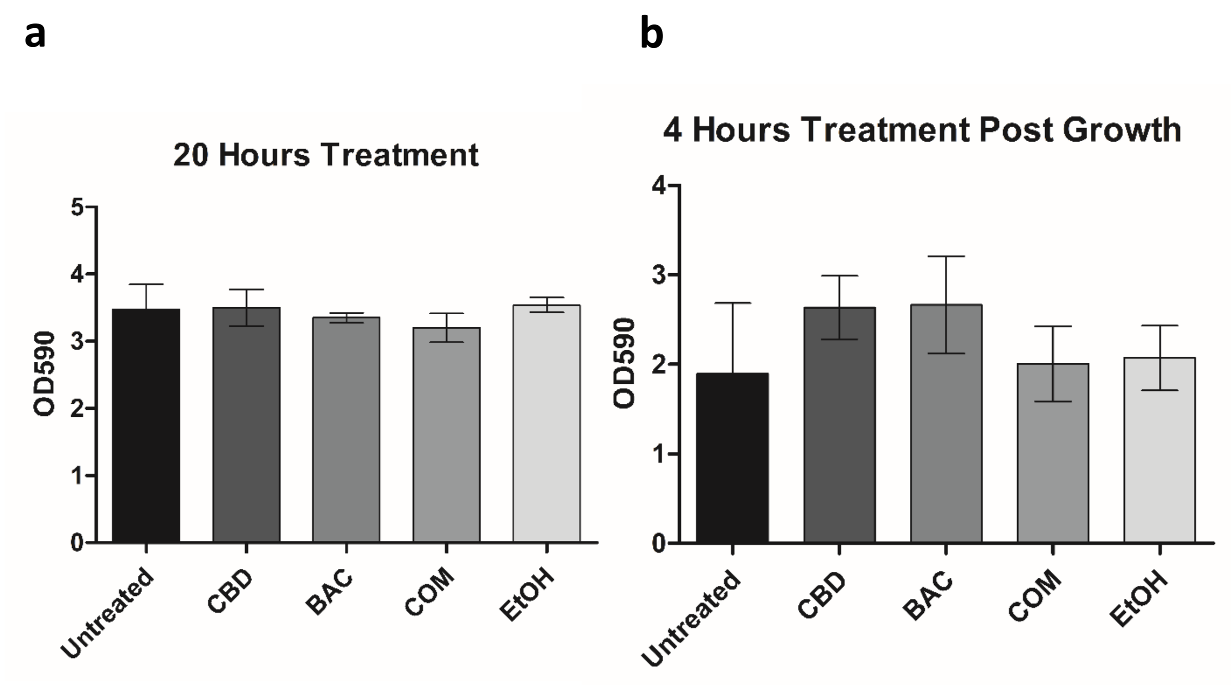

Figure S9: Static biofilm on silicone discs performed as described below. The cells were either treated with cannabidiol (CBD) and/or bacitracin (BAC) at time 0 for 20 hours (a) to discover effects on biofilm formation or left to form biofilm for 20 hours before treatment for 4 hours to discover effects on biofilm degradation (b). No statistical differences between the treatments were observed using one-way ANOVA with Bonferroni's Multiple Comparison Test.

Biofilm quantification using crystal violet stain

An ON culture of USA300 was diluted to OD600 0.1 in PBS and further diluted 20x in PBS containing 10% plasma. 1.5 mL of the culture was added to a 24-well plate containing a sterile silicone discs (LEBO Production AB) in each well with a thickness of 2 mm and a shore A hardness of 60. The cells were either treated at time 0 for 20 hours or left to form biofilm for 20 hours before treatment for 4 hours using either 1 µg/mL CBD, 16 µg/mL BAC, the combination of CBD and BAC, EtOH, or left untreated. The plates were incubated at 37 degrees with agitation. After treatment the liquid was carefully removed, and the cells were stained for 15 minutes using 4% crystal violet. After staining, the excess crystal violet was removed by pipetting and the remaining crystal violet removed by PBS washing. The silicone discs were moved to a clean 24-well plate and the crystal violet bound to the biofilm was removed using 33% acetic acid. The amount of biofilm was quantified by measuring optical density at 590 nm.
